# A Review of Auraptene as an Anticancer Agent

**DOI:** 10.3389/fphar.2021.698352

**Published:** 2021-06-22

**Authors:** Zahra Tayarani-Najaran, Nilufar Tayarani-Najaran, Samira Eghbali

**Affiliations:** ^1^ Medical Toxicology Research Center, Mashhad University of Medical Sciences, Mashhad, Iran; ^2^ Targeted Drug Delivery Research Center, Pharmaceutical Technology Institute, Mashhad University of Medical Sciences, Mashhad, Iran; ^3^ Department of Prosthodontics, School of Dentistry, Mashhad University of Medical Sciences, Mashhad, Iran; ^4^ Department of Pharmacognosy, School of Pharmacy, Birjand University of Medical Sciences, Birjand, Iran; ^5^ Cellular and Molecular Research Center, Birjand University of Medical Sciences, Birjand, Iran

**Keywords:** Auraptene, Rutaceae, cytotoxicity, anticancer activity, cancer cells

## Abstract

Auraptene is a bioactive monoterpene coumarin isolated from *Citrus aurantium* and *Aegle marmelos* that belong to the Rutaceae family. Auraptene can modulate intracellular signaling pathways that control cell growth, inflammation and apoptosis and can exert pharmacological properties such as anti-bacterial, anti-fungal, antileishmania and anti-oxidant activity. Auraptene had inhibitory and chemo-preventive effects on the proliferation, tumorigenesis and growth of several cancer cell lines through increase in the activity of glutathione S-transferase, formation of DNA adducts and reduction of the number of aberrant crypt foci. Auraptene exhibits anticancer effects via targeting different cell signaling pathways such as cytokines, genes modulating cellular proliferation, growth factors, transcription factors and apoptosis. The present review is a detailed survey of scientific researches on the cytotoxicity and anticancer activity of Auraptene on cancer cells and tumor bearing animals.

## Introduction

Coumarins are a large class of natural compounds mainly found in the Apiaceae, Rutaceae, and Composita families. The three main groups of coumarins are: 1) ring-fused coumarins, 2) substituted coumarins and 3) C- and O-prenylcoumarins (Eghbaliferiz et al., 2020).

**FIGURE 1:**
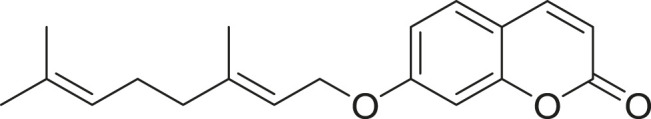
Structure of auraptene.

Auraptene, 7-geranyloxycoumarin, is a member of umbelliferone coumarins in which the phenolic hydrogen has been replaced by a geranyl group. Auraptene is isolated from many of the edible fruits and vegetables of the genus *Ferula* and *Citrus* like lemons, grapefruits and oranges. Auraptene indicates a variety of therapeutic properties such as antidiabetic, antiprotozoal, anti-bacterial, anti-fungal, anti-genotoxic, antileishmanial, anti-inflammatory, antihelicobacter, and immunomodulatory activities (Bibak et al., 2019). This compound showed significant effect on the treatment of several chronic illnesses including hypertension, nonalcoholic fatty liver and cystic fibrosis (Derosa et al., 2016; Figure 1).

Tumor is a group of cells/tissues due to the activation of various oncogenes or inactivation of tumor suppressors has lost its control on the normal growth at the gene level. Tumor tissue can be divided into malignant (cancerous) and benign (non-cancerous) according to the size and growth features. Malignant tumor grows rapidly and often infiltrate to the surrounding tissues without envelops on the surface. Patients with advanced cancer after surgical excision exhibited severe systemic symptoms and high recurrence rate, that causing a big challenge for cancer therapy. In view of the enormous damage caused by cancer, the development of new antitumor treatments has become a research hotspot. Natural products are a good source of antitumor compounds. In recent years, auraptene have drawn scientists’ attention because of its broad-spectrum and effective antitumor activities (Fu et al., 2020).

The information and literature available in this review have been obtained through the Pubmed, Scopus, and Science Direct databases for English articles published until 2020. For this purpose, we used appropriate keywords including “auraptene” or “coumarin” and “cancer” or “cytotoxic.” The purpose of this review is to summarize the therapeutic effect and mechanism of auraptene in different types of cancers.

## Anticancer Properties of Auraptene

**FIGURE 2:**
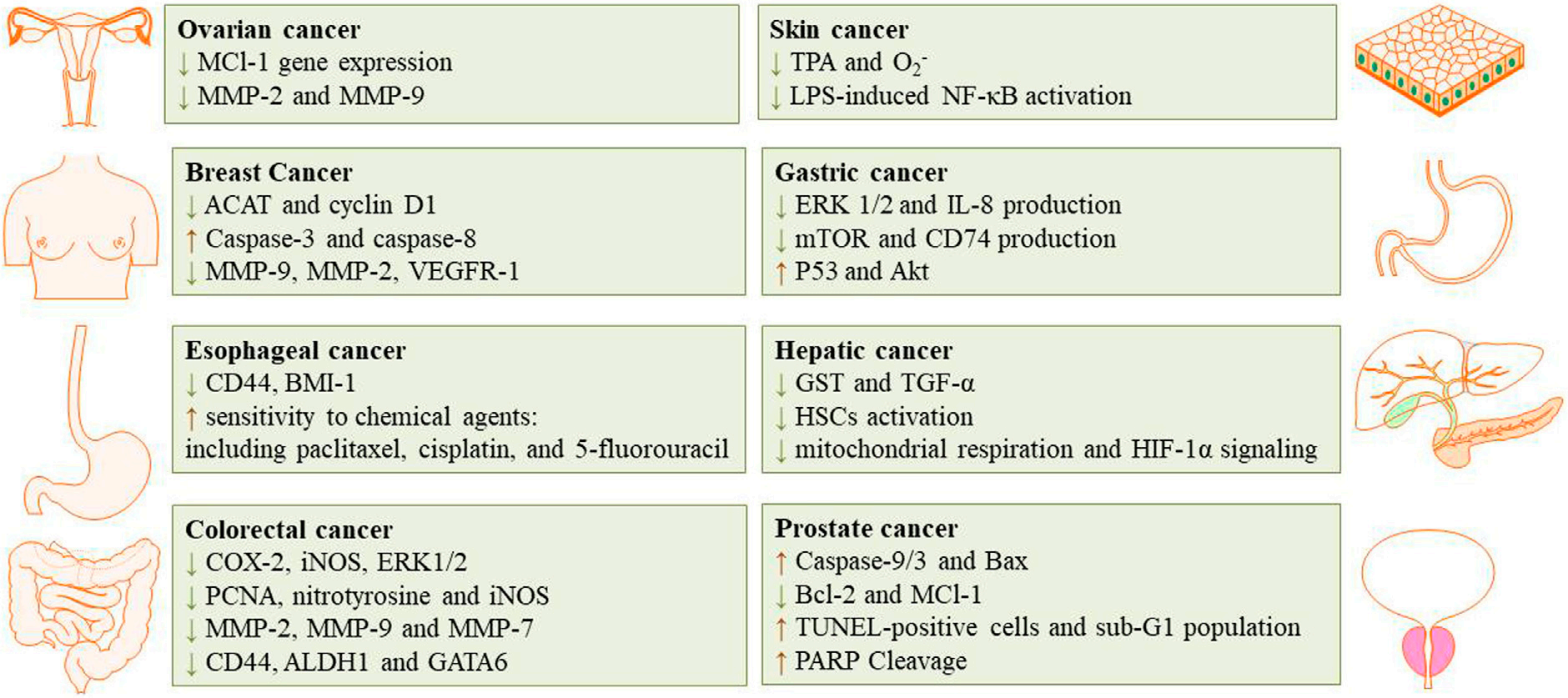
The inhibition effects of auraptene in different type of cancer.

**FIGURE 3:**
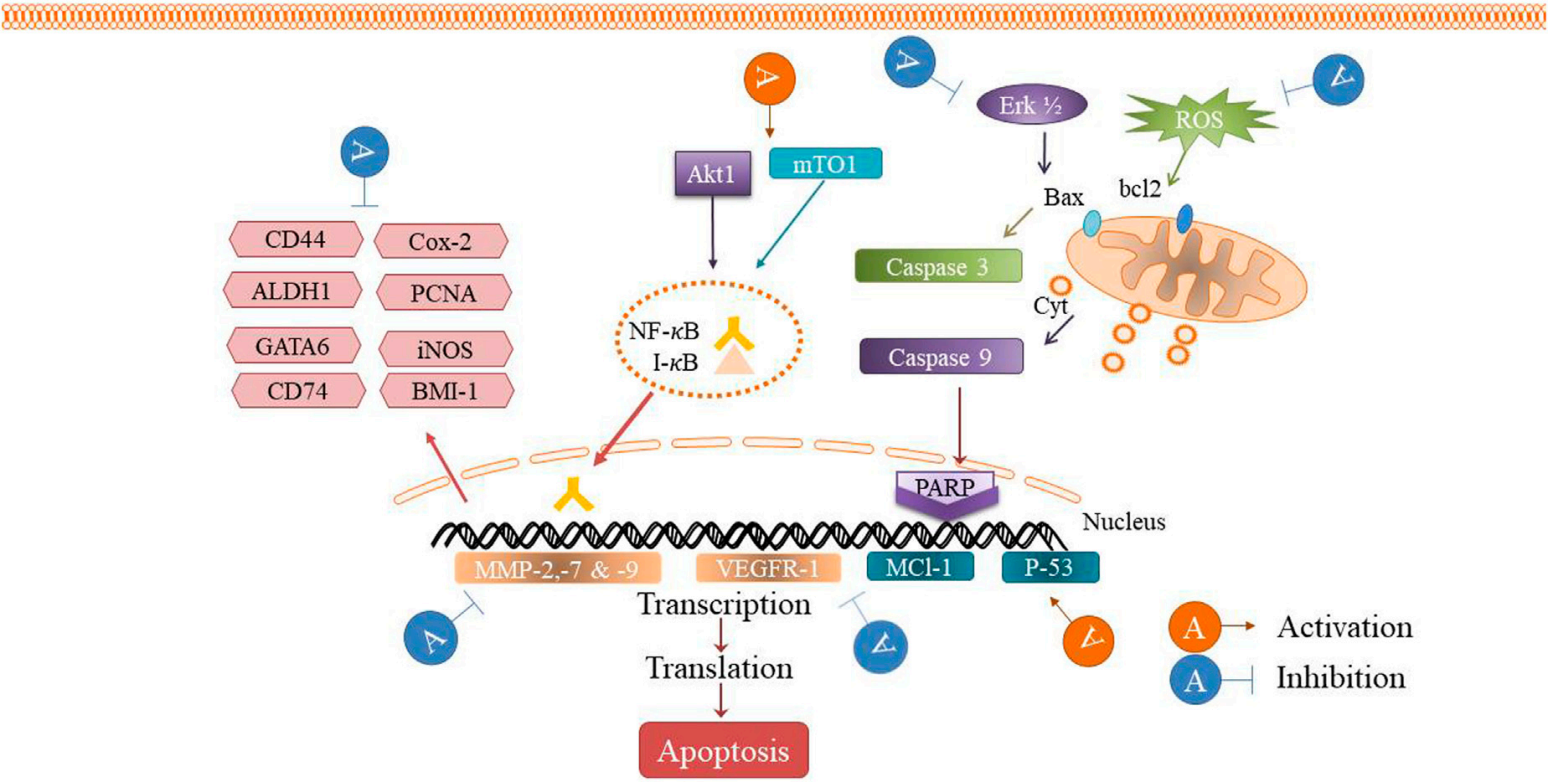
The antitumor mechanism of auraptene.

The cytotoxic, antitumor and anticancer activities of auraptene have been addressed in many *in vivo* and *in vitro* studies. The underling mechanisms have been discussed in the next paragraphs and summarized in Figure 2 and Table 1 and Figure 3 displays the molecular mechanism of auraptene in cancer treatment.

**TABLE 1:** The antitumor experiment of auraptene *in vitro* and *in vivo*.

Type of cancer	Experimental model	Dose	IC_50_	Mechanism	References
Breast cancer	SW-620, MDA-MB-231, MCF-7	0.1–100 µM	4.18 μg/ml	Modulation of ERs and suppression of acyl-CoA	De Medina et al. (2010)
Breast cancer	MDA-MB-231, MCF-7 and rat	1–50 µM 200, 500 ppm	85% inhibition	Inhibition of cyclin D1	Krishnan et al. (2009)
Breast cancer	MCF-7	250 μg/ml	61.3 μg/ml	Up-regulation of caspase-3 and caspase-8 and down-regulation of MMP9, MMP2, VEGFR-1, and VEGFR-2	Charmforoshan et al. (2019)
Breast cancer	MCF-7	10, 20, 40 μg/ml	17.26–29.66 μg/ml	Down-regulation of Mcl-1 mRNA	Motlagh and Gholami (2017)
Breast cancer	MCF-7 cells	500 ppm		Reduction of cyclin D1 protein expression and inhibition of IGF-1	Krishnan and Kleiner-Hancock (2012)
Gastric cancer	MGC-803 cells	0–4 µM	0.78–10.78 µM	Expression of apoptosis- related protein	Li et al. (2018)
Gastric cancer	C57BL/6 mice	100, 500 ppm		Inhibition of CD74 production	Sekiguchi et al. (2012)
Gastric cancer	NCI-N87 cells	20 µM		Reduction of ERK 1/2 activation and IL-8 production	Sekiguchi et al. (2010)
Gastric cancer	SNU-1 cells	25–200 μg/ml	≤25 µM	Inhibition of mTOR, activation of p53 and increase in the phosphorylation of Akt	Moon et al. (2015)
Colon cancer	HT-29 and HT-116 cells	10 µM	≥50%	Suppression of CD166 and CD44 and inhibition of colonospheres	Epifano et al. (2013)
Colon cancer	F344 rats	100, 500 ppm		Activation of the phase II enzymes QR and GST	Tanaka et al. (1998a); Tanaka et al. (1997)
Colon cancer	C57BL/KsJ-db/db mice	10 mg/kg		Inhibition of COX-2 and iNOS, reduction of cell proliferation and lipid profiles	Hayashi et al. (2007)
Colon cancer	C57BL/KsJ-db/db mice	10 mg/kg	67–80% inhibition	Reduction of triglycerides and anti-inflammatory activity of auraptene	Tanaka et al. (2008)
Colon cancer	CD-1 (ICR) mice	100, 500 ppm	63–83% inhibition	Suppression of colonic inflammation and modulation of proinflammatory cytokines	Tanaka et al. (2010)
Colon cancer	HT-29 cell line	1–50 µM	2.8 and 3 µM	Suppression of proMMP-7 and inhibition of ERK1/2	Kawabata et al. (2006b)
Colon cancer	ICR mice	100, 500 ppm		Reduction of COX-2, PCNA, iNOS	Kohno et al. (2006)
Colon cancer	Colonic mucosa mouse	0.1% w/w	82% inhibition	Inhibition of MMP-2, MMp-9 and suppression DSS	Kawabata et al. (2006a)
Colon cancer	HT29 cells	10, 20 μg/ml	39 μg/ml	Reduction of hyperthermia and down-regulation of HSP27	Moussavi et al. (2018)
Colon cancer	HT29 cells	10, 20, 40, and 80 μg/ml	31.8–42.1%	Down regulation of CD44, ALDH1 and inhibited expression of GATA6	Moussavi et al. (2017)
Hepatic cancer	F344 rats	100, 500 ppm	83% inhibition	Reduction of GST, TGF-α	Sakata et al. (2004)
Hepatic cancer	F344 rats	100, 500 ppm		β-catenin mutation	Hara et al. (2005)
Hepatic cancer	C57BL/6 mice	30 mg/kg		Reduction of toxic bile acids, inhibition of inflammation and HSCs activation	Gao et al. (2018)
Hepatic cancer	Rat	500 ppm		Nob induction of cell cycle inhibition and apoptosis	Ohnishi et al. (2004)
Hepatic cancer	RCC4 cell line	0–100 μM		Inhibition the mitochondrial respiration and blockade HIF-1α	Jang et al. (2015)
Prostate cancer	PC3 and DU145	30, 60 μM	30–45%	Activation of caspase-9/3, Bax, inhibition of Bcl-2 and Mcl-1, increase the TUNEL-positive cells	Lee et al. (2017)
Prostate cancer	PC3 and DU145	500 ppm		Induction of apoptosis and cell cycle arrest	Tang et al. (2007)
Skin cancer	ICR mouse skin	18 μM	450 μM	Inhibition of TPA and suppression of O_2_^−^	Murakami et al. (1997)
Skin cancer	C57BL/6 mice	500, 1,000 mg/kg		Induction of apoptosis, inhibition of metastasis of B16BL6 melanoma cells	Tanaka et al. (2000)
Skin cancer	Xenograft mouse	200 mg/kg	84% inhibition	Suppression of LPS-induced NF-κB activation	Kleiner-Hancock et al. (2010)
Skin cancer	M4Beu melanoma	10 μg/ml	17.1 µM	Induction of caspase-dependent apoptosis and cell-cycle arrest	Barthomeuf et al. (2008)
Ovarian cancer	Hela cell line	10, 20, 40 μg/ml	13.33–13.87 μg/ml	Down-regulation of MCl-1 gene expression	Motlagh and Gholami (2017)
Ovarian cancer	Hela and A2780 cell line	12.5–100 μM	31.49 and 47.93 μM	Reduction of MMP-2 and MMP-9 enzymatic activity	Jamialahmadi et al. (2018)
Esophageal cancer	KYSE30 cells	20 μg/ml	76–80 μg/ml	Reduction expression of CD44, BMI-1	Saboor-Maleki et al. (2017)
Esophageal cancer	KYSE30 cells	10, 20, 40 μg/ml	11.75–15.25 μg/ml	Down-regulation of Mcl-1 gene expression	Motlagh and Gholami (2017)
Leukaemia	Jurkat cells	20 μg/ml	16.5 μg/ml	Activation of caspase cascade, caspase-8 and caspase-3, degradation of PARP and suppression of Bcl-xL	Jun et al. (2007)
Leukaemia	Jurkat cells	10, 20, 40 μg/ml	11.3–11.49 μg/ml	Down-regulated Mcl-1 mRNA expression	Motlagh and Gholami (2017)
Oral carcinogenesis	F344 rats	100, 500 ppm	63–91% reduction	Suppression of 4-NQO activity, decreased dysplastic lesions, inhibited the expression of cell proliferation	Tanaka et al. (1998b)

### Breast Cancer

Breast cancer is the most widespread and second leading cause of death in the female adult population. Hormone positive and negative Breast cancer may be treated according to the hormone status of the cancer cells. Hormone suppressive treatment is assigned to hormone sensitive cells (EghbaliFeriz et al., 2018).

Auraptene, a prenyloxycoumarin from *Citrus* species, demonstrated anticancer activity against breast cancer. The underlying mechanism responsible for this effect is the modulation of estrogen receptors (ERs) and suppression of cholesterol acyl transferase (ACAT) (De Medina et al., 2010). Auraptene inhibited the proliferation of MDA-MB-231 and MCF-7 human breast cancer cells and significantly inhibited cyclin D1 dose-dependently and delayed tumor progression in rat mammary tumors (Krishnan et al., 2009). It has also been reported that the dietary administration of auraptene delayed tumor expansion (or extension). Auraptene reduced cyclin D1 protein expression and inhibition of insulin-like growth factor-1(IGF-1) in MCF-7 cells (Krishnan and Kleiner-Hancock, 2012). In another study, auraptene from *Ferula szowitsiana* root indicated anti-tumor activity in human breast cancer cells via up-regulation of caspase-3 and caspase-8 and down-regulation of MMP-9, MMP-2, VEGFR-1 (vascular endothelial growth factor), and VEGFR-2 genes (Charmforoshan et al., 2019). Motlagh et al. investigated the cytotoxic effects of umbelliprenin and auraptene against MCF-7 cell lines. Auraptene was more cytotoxic than umbelliprenin and exerted this effect through down-regulation of Myeloid Cell Leukaemia Type-1 (Mcl-1) mRNA expression (Motlagh and Gholami, 2017).

### Colorectal Cancer

Colorectal cancer is one of the most prevalent and third leading cause of death among patients in the world. The best strategy for treatment of colorectal cancer is surgical resection combined with chemotherapy and radiation (Kaur and Kaur, 2015).

Auraptene at a concentration of 10 µM significantly inhibited the growth and formation of HT-29 and HT-116 cells *in vitro*. Auraptene was reported to suppress the expression of CD166 and CD44 in chemo-resistant HT-29 cells and inhibit the formation of colonospheres and growth of both chemo-resistant and wild-type colon cancer cells (Epifano et al., 2013). The administration of auraptene significantly inhibited the development of azoxymethane (AOM)-induced colonic aberrant crypt foci (ACF) in male F344 rats. The mechanisms responsible for this effect were activation of the phase II enzymes QR (quinone reductase) and GST (glutathione Stransferase), reduction of lipid peroxidation and suppression of cell proliferation biomarkers such as ornithine decarboxylase activity, 5-bromo-29-deoxyuridine labeling-index and polyamine content in the colonic mucosa (Tanaka et al., 1997; Tanaka et al., 1998a). In another study, administration of auraptene (10 mg/kg) significantly inhibited the incidence of colon carcinogenesis in C57BL/KsJ-db/db mice. The inhibition of COX-2 and iNOS in colonic epithelial cells, reduction of cell proliferation, lipid profiles and induction of apoptosis was responsible for this effect (Hayashi et al., 2007). Also, the incidence and multiplicity of colonic adenocarcinomas is suppressed by administration of collinin and auraptene at dose of 0.05 and 0.01%, respectively in clitis- and obesity-related colon tumorigenesis models in C57BL/KsJ-db/db mice. This activity attributed to anti-inflammatory activity of auraptene and collinin (Tanaka et al., 2008). Auraptene has been reported to prevent the proliferation of colorectal cancer by modulation of inflammatory and expression of proinflammatory cytokines in CD-1 (ICR) mice. These results indicate that the administration of auraptene suppresses the colonic inflammation and modulates proinflammatory cytokines including interleukin (IL)-6 and IL-1b, tumor necrosis factor-a, nuclear factor-kappaB, Stat3 and NF-E2-related factor 2 in adenocarcinomas (Tanaka et al., 2010). In a study conducted by Kawabata et al., auraptene suppressed the growth of human colorectal adenocarcinoma HT-29 cell line. The suppression of proMMP-7 (Matrix metalloproteinase) production and inhibition of extracellular signal-regulated kinase (ERK1/2) protein translation responsible for this effect (Kawabata et al., 2006b).

Kohno et al. reported that auraptene significantly reduced the rates of cyclooxygenase (COX)-2, proliferating cell nuclear antigen (PCNA), nitrotyrosine and inducible nitric oxide (iNOS) in adenocarcinomas. Also, auraptene increased the apoptotis and suppressed the occurrence of colonic adenocarcinoma in male ICR mice (Kohno et al., 2006). It addition, auraptene inhibited MMP-2, MMP-9 expression in HT-29 human colon cancer cells suggesting the anti-metastatic activity of auraptene (Kawabata et al., 2006a). In another study, auraptene reduced the toxicity of hyperthermia in human colon adenocarcinoma HT29 cells as a new and important therapeutic strategy usually used as an adjunct to cancer treatment, which was verified by down-regulation of HSP27 (Moussavi et al., 2018). Jalilzadeh et al. reported that nano-encapsulated formulations with biodegradable penta-block PLA-PCL-PEG-PCL-PLA and triblock PCL-PEG-PCL increase the bio-distribution, solubility and delivery of aurapetne to targeted sites and improve the anticancer activity of auraptene especially in colon cancer cells (Jalilzadeh et al., 2020). In colon adenocarcinoma cells, synergism between auraptene and ionizing radiation increased the efficacy of treatment on HT29 cells. Auraptene down regulated CD44, ALDH1 (aldehyde dehydrogenase 1) and inhibited the expression of GATA6 (GATA binding protein 6) (Moussavi et al., 2017). Coadministration of auraptene and radiotherapy for treatment of colon carcinoma cells was investigated *in vitro* and *in vivo.* Increase in radiotherapy effects by AUR was observed and confirmed by increased number of apoptotic cells. *In vivo*, after administration of AUR + radiotherapy significant regression in tumor size, down regulation of Cyclin D1 and CD44, involvement of PI3K-AKT-mTORC signaling pathway and Caspase-3 was observed (Salari et al., 2020).

### Gastric Cancer

The one of the most common malignancies and third leading cause of death worldwide is gastric cancer. The main therapeutic approach for the treatment of gastric cancer is chemotherapy (Torre et al., 2015).

The major human pathogen that plays an important role in gastric cancer and chronic gastritis is *Helicobacter pylori*. Auraptene inhibited *Helicobacter pylori* and CD74 production by reduction of extracellular signaling-regulated kinase (ERK) 1/2 activation and IL-8 production in NCI-N87 gasteric carcinoma cells (Sekiguchi et al., 2010). It has also been reported that auraptene suppresses *Helicobacter pylori* adhesion. The mechanism attributed to this activity was inhibition of CD74 production and reduction of inflammatory cytokine expression such as tumor necrosis factor-α and interleukin-1β in C57BL/6 mice (Sekiguchi et al., 2012). In another study auraptene, indicated anticancer effect against SNU-1 gastric cancer cells. The underlying mechanism was induction of apoptosis and cell cycle arrest by inhibition of mTOR signaling pathway and activation of p53 along with increase in the phosphorylation of Akt (Moon et al., 2015). Li et al. reported that four new terpene coumarins with notable changes in the skeletal backbone from 2-Z auraptene, a synthesized monoterpene coumarin, demonstrate antiproliferative activity against human gastric cancer cells (MGC-803) with IC_50_ values of 0.78 ± 0.13–10.78 ± 1.83 µM and induce apoptosis (Li et al., 2018).

### Hepatic Cancer

As one of the most common types of liver cancer, Hepatocellular carcinoma (HCC) is seen most often in people suffering from chronic liver diseases such as hepatitis B or hepatitis C virus infected individuals. Plant based natural products are routinely used as adjuvants to chemotherapeutics (Yang et al., 2019).

Auraptene was introduced as a powerful chemopreventive agent against N,N-diethylnitrosamine (DEN) that initiated hepatocarcinogenesis in male F344 rats. In one study, the consumption of AUR at doses of 100 and 500 ppm during DEN exposure reduced the numbers of glutathione S-transferase (GST) and transforming growth factor (TGF)-α and also suppressed the incidence of liver cell carcinoma (Sakata et al., 2004). It has also been reported that daily consumption of auraptene during DEN exposure inhibited hepatocellular carcinoma *via* β-catenin mutation in male F344 rats (Hara et al., 2005). Gao et al. investigated the hepatoprotection of auraptene against thioacetamide (TAA)-induced hepatic fibrosis in mice. The underlying mechanism was reduction of toxic bile acids, inhibition of inflammation, and activation of hepatic stellate cells (HSCs), all of which were related to the activation of farnesoid X receptor (FXR) (Gao et al., 2018). It seems inhibition of HCC cell growth and cell cycle arrest are not the mechanisms of action of auraptene in hepatocellular carcinoma of rat when compared with nobiletin (Ohnishi et al., 2004). In another study which investigated F344 rats, similar data was obtained and auraptene did not inhibit cell proliferation (Kitano et al., 2000).

Hypoxia-inducible factor 1α (HIF-1α) is an important regulator of cancer metabolism, angiogenesis and migration which is produced in renal cell carcinoma (RCC). Jang et al., reported auraptene suppresses the progression of RCC through inhibition of mitochondrial respiration and blockade of HIF-1α signaling, without any cytotoxic effects (Jang et al., 2015).

### Prostate Cancer

As the main leading cause of death among men, prostate cancer is routinely treated with chemotherapy, hormonal therapy and radiotherapy. Some natural products have shown promising effects as adjuvant treatments for prostate cancer (Lee et al., 2017).

Lee et al. investigated the anticancer mechanism of auraptene in PC3 and DU145 prostate cancer cells. Auraptene induces apoptosis in prostate cancer cells through activation of caspase-9/3 and Bax, inhibition of Bcl-2 and myeloid cell leukemia 1 (Mcl-1), increases in the number of transferase dUTP nick end labeling (TUNEL)-positive cells and sub-G1 population, and regulation of apoptosis-related proteins such as poly (ADP-ribose) polymerase (PARP) (Lee et al., 2017). In another study, the growth of androgen positive DU145 and PC3 human prostate cancer cells was inhibited by both nobiletin and auraptene in a dose-dependent manner via induction of apoptosis and cell cycle arrest (Tang et al., 2007).

### Skin Cancer

Melanoma and nonmelanoma are two main classes of skin cancer with high and increasing incidence in the world. Surgery freezing (cryotherapy), anti-cancer creams, radiotherapy and a form of light treatment called photodynamic therapy (PDT) are therapeutic choices for skin cancer (Taleghani et al., 2021).

In ICR mouse skin, auraptene suppressed the skin tumorgenesis through inhibition of 12-O-tetradecanoylphorbol-13-acetate (TPA) and suppression of superoxide (O_2_^−^) and intracellular hydroperoxide production in leukocytes (Murakami et al., 1997). In another study, auraptene and 1,4-phenylenebis (methylene) selenocyanate (p-XSC) suppressed the metastasis of melanoma cells to lung in mice. The underlying mechanism was induction of apoptosis and inhibition of metastasis of B16BL6 melanoma cells to lung (Tanaka et al., 2000). In human squamous cell carcinoma (SCC) xenografts, auraptene increased the protective effect of all-trans retinoic acid (ATRA) against human skin cancer cell growth in female SCID/bg mice and suppressed the LPS-induced NF-κB activation (Kleiner-Hancock et al., 2010). Barthomeuf et al. reported that auraptene inhibits the growth of human M4Beu melanoma cells (IC_50_ 17.1 µM) via induction of caspase-dependent apoptosis and cell-cycle arrest in G1 (Barthomeuf et al., 2008).

### Ovarian Cancer

Cervical and ovarian cancers are among the most common gynecologic cancers. Surgery, chemotherapy, and radiation are among the treatment plans (Koh et al., 2015). Like other cancers, herbs and phytocompounds are being progressively introduced as efficient complementary treatments for gynecologic cancers.

Between umbelliprenin and auraptene, auraptene showed more cytotoxic effects against Hela cancer cells *via* down-regulation of MCl-1 gene expression (Motlagh and Gholami, 2017). Auraptene has been reported to decrease the viability of human cervical and ovarian cancer cells and suppresses the migration and invasion of Hela and A2780 cell line by reduction of matrix metalloproteinase-2 (MMP-2) and metalloproteinase-9 (MMP-9) enzymatic activity, respectively (Jamialahmadi et al., 2018). Maleki et al. reported that prenylation at position six of the coumarin ring significantly improved the anticancer activity of aurapten. In this study, eight coumarins were examined and among them umbelliprenin, auraptene, umbelliferone and herniarin with prenylation substitution at position six indicated the best anticancer activity, especially against cervical cancer, with minimal cytotoxicity on normal cells (Maleki et al., 2020).

### Esophageal Cancer

Esophageal cancer is the seventh common cancer in humans. In a study conducted by Saboor-Maleki et al., auraptene upregulated P53 and P21 and downregulated the expression of stem-like cancer cell markers such as CD44 (cluster of differentiation 44), BMI-1 (B cell-specific moloney murine leukemia virus integration site 1) and increased the sensitivity of esophageal squamous cell carcinoma (ESCC) to paclitaxel, cisplatin, and 5-fluorouracil (Saboor-Maleki et al., 2017). In another study on esophageal carcinoma cell line (KYSE-30), auraptene down-regulated the MCl-1 gene expression and indicated more cytotoxic effect in comparison with umbelliprenin (Motlagh and Gholami, 2017).

### Other Cancer

Jun et al., showed that auraptene, from leaves of *Z. schinifolium,* on Jurkat T cells induce apoptosis through activation of caspase cascade (caspase-8 and caspase-3), degradation of PARP and suppression of Bcl-xL (Jun et al., 2007). In comparison between cytotoxic effects of auraptene and umbelliprenin on Jurkat cells, auraptene was more cytotoxic than umbelliprenin and down-regulated Mcl-1 mRNA expression (Motlagh and Gholami, 2017).

In male F344 rats, dietary administration of auraptene suppressed the carcinogenic activity of 4-nitroquinoline 1-oxide (4-NQO) and inhibited development of oral neoplasms, increased the activity of gluthathione S-transferase (GST) and quinone reductase (QR) in the tongue and liver and decreased dysplastic lesions (Tanaka et al., 1998b).

## Pharmacokinetics of Auraptene

The pharmacokinetics of auraptene has been evaluated in several studies. Ye et al. determined the pharmacokinetics of auraptene in rat plasma using LC-MS/MS method. Data showed that a dose range of 0.5–200 ng/ml of auraptene was safe and bioavailability of oral administration of auraptene was merely 8.5% in rats (Ye et al., 2016). In another study, the absorption and metabolism of auraptene in rodent models was examined. The result indicated that oral administration of AUR at 50–200 mg/kg body wt induced glutathione S-transferase (GST) activity and xenobiotic phase II enzymes, and indicated chemopreventive effects in rodents. These effects increase, absorption and stable (stabilize) localization in the colon and liver (Murakami et al., 2000). Auraptene showed higher absorption rate in comparison with 7-ethoxycoumarin, with longer life span due to the presence of the geranyloxyl side chain (Kuki et al., 2008).

## Toxicology

The acute toxicity of orally administrated auraptene in rats was investigated and different concentrations of the compound (125–2,000 mg/kg body weight) had no effect on mortality. However, administration of auraptene demonstrated some differences in ALP, ALT, AST, blood urea, total bilirubin, total protein, haematocrit, hemoglobin, RBC count, platelet count and MCHC (mean corpuscular haemoglobin concentration) in AUR-treated animals as compared to the controls but all were in normal reference ranges. Histopathological examination of organs such as liver, kidneys, bone marrow, heart and lungs indicated no toxic effects of auraptene and showed the safety of the compound (Vakili et al., 2017).

## Conclusion

This review evaluated the effects of auraptene on different cancer cells both *in vivo* and *in vitro*. Auraptene had inhibitory and chemo-preventive effects on the proliferation, tumorigenesis and growth of several cancer cell lines through increase in the activity of glutathione S-transferase, formation of DNA adducts and reduction of the number of aberrant crypt foci. Auraptene exhibits anticancer effects via targeting different cell signaling pathways such as cytokines, genes modulating cellular proliferation, growth factors, transcription factors and apoptosis.

Modulation of Bcl-2, increase in amount of Bax protein, reduction of MMP-2 and MMP-9, inhibition of NF-κB, activation of caspase and p53-independent, suppression of superoxide (O_2_^−^), down-regulation of Mcl-1 mRNA expression and reduction of mitochondrial membrane potential are important mechanisms for the cytotoxic and anticancer effects of auraptene. Low cytotoxic IC_50_s values of auraptene demonstrated its potency as a worthy phytochemical for cancer treatment. However, lack of clinical evaluation has made it difficult to use auraptene in conventional chemotherapeutic regimens for cancer treatment.

In conclusion, auraptene has an excellent safety profile and the ability to affect multiple molecular targets which are important in the prevention and/or management of a number of cancers. However, further studies and more clinical trials are needed to fully elucidate the putative potential of auraptene as an effective anticancer agent.

## Glossary

ACAT: cholesterol acyltransferase
ACF: aberrant crypt foci
ALDH1: aldehyde dehydrogenase 1
AOM: azoxymethane
ATRA: all-trans retinoic acid
AUR: auraptene
BMI-1: B cell-specific moloney murine leukemia virus integration site 1
CD44: cluster of differentiation 44
COX-2: cyclooxygenase-2
ERs: estrogen receptors
ERK1/2: extracellular signal-regulated kinase
ESCC: esophageal squamous cell carcinoma
GATA6: GATA binding protein 6
GST: gluthathione S-transferase
HIF-1α: hypoxia-inducible factor 1α
IGF-1: insulin-like growth factor-1
iNOS: inducible nitric oxide synthase
MMP9: matrix metallopeptidase 9
MMP2: matrix metallopeptidase 2
Mcl-1: myeloid cell leukaemia Type-1
NF-κB: nuclear factor-kappaB, Stat3
NF-E2: nuclear factor erythroid 2
4-NQO: 4-nitroquinoline 1-oxide
PARP: poly (ADP-ribose) polymerase
PCNA: proliferating cell nuclear antigen
PDT: photodynamic therapy
QR: quinone reductaseand
RCC: renal cell carcinoma
SCC: squamous cell carcinomas
TNF-α: tumor necrosis factor-a, signal transducer and activator of transcription 3
TPA: tetradecanoylphorbol-13-acetate
TUNEL: transferase dUTP nick end labeling
VEGFR-1: Vascular endothelial groth factor
